# *Egr1* Gene Expression as a Potential Biomarker for In Vitro Prediction of Ocular Toxicity

**DOI:** 10.3390/pharmaceutics13101584

**Published:** 2021-09-29

**Authors:** Da-Bin Hwang, Shin-Young Kim, Dong-Hoon Won, Changuk Kim, Yoo-Sub Shin, Jong-Hwan Park, Young-Jin Chun, Kyung-Min Lim, Jun-Won Yun

**Affiliations:** 1 Department of Biotechnology, The Catholic University of Korea, Bucheon 14662, Korea; hmodabin@naver.com (D.-B.H.); asdg425@naver.com (S.-Y.K.); wdonghooni@hanmail.net (D.-H.W.); a0223asd@naver.com (C.K.); yousubbabo@naver.com (Y.-S.S.); 2 Laboratory Animal Medicine, College of Veterinary Medicine, Chonnam National University, Gwangju 61186, Korea; jonpark@jnu.ac.kr; 3 College of Pharmacy, Chung-Ang University, Seoul 06974, Korea; yjchun@cau.ac.kr; 4 College of Pharmacy, Ewha Womans University, Seoul 03760, Korea; 5 Department of Medical and Biological Sciences, The Catholic University of Korea, Bucheon 14662, Korea

**Keywords:** early growth response-1, eye irritation, biomarker, reporter vector system, immortalized corneal cell

## Abstract

Animal models are used for preclinical toxicity studies, and the need for in vitro alternative methods has been strongly raised. Our study aims to elucidate the potential mechanism of change in EGR1 expression under situations of toxic injury and to develop an *Egr1* promoter–luciferase gene reporter assay for an in vitro alternative method for toxicity prediction in drug discovery. We first found an increase in early growth response-1 (EGR1) mRNA/protein expressions in the liver and kidney of cisplatin-treated injured rats. Additionally, the EGR1 protein level was also elevated under situations of ocular injury after sodium lauryl sulfate (SLS) eye drops. These in vivo observations on injury-related EGR1 induction were confirmed by in vitro studies, where human corneal epithelial cells were treated with representative irritants (SLS and benzalkonium chloride) and 17 chemicals having different UN GHS irritant categories. Additionally, our results suggest the involvement of ERK, JNK, p38 MAPK pathways in EGR1 elevation in response to gamma-butyrolactone-induced injury. As EGR1 is considered to be a pivotal factor in proliferation and regeneration, siRNA-mediated knockdown of *Egr1* promoted cytotoxic potential through a delay of injury-related recovery. More importantly, the elevation of promoter activities was observed by various irritants in cells transfected with *Egr1* promoter-reporter vector. In conclusion, *Egr1* can be a potential biomarker in a promoter-reporter system to improve the accuracy of in vitro predictions for ocular irritation.

## 1. Introduction

As animal models share similar biological pathways with humans, preclinical pharmacology and toxicology testing must be performed inevitably on these models before the clinical phase on humans for approval by regulatory agencies [1]. In particular, as an essential part of a comprehensive toxicity program in the processes of drug discovery and development, the degree of eye and skin irritation for new chemicals, drugs, and cosmetics should be measured before exposure to humans [2]. For this, the Draize rabbit eye irritation test has been commonly used, in which chemicals are applied directly into the rabbit’s eyes to observe changes in eye state [3,4]. However, ethical issues, including considerable pain and discomfort, have been raised about the use of laboratory animals, leading to the development of in vitro alternatives to replace animal experiments according to the 3Rs (reduction, refinement, and replacement) principle [5,6].

One of the most important in vitro alternative tests is the hen’s egg test–chorioallantoic membrane test. This test evaluates the blood vessel reaction of the chorioallantoic membrane after exposure to liquid or solid test materials using fertilized chicken eggs [7,8]. The bovine cornea opacity/permeability assay using a by-product (corneas) from freshly slaughtered cattle [9], the isolated chicken eye test [10], and rabbit cornea epithelium (RCE) model [11] were also developed for evaluation of the in vitro eye irritation potential of various chemicals, such as pharmaceuticals, cosmetics, and their raw materials. Various 3D reconstructed human cornea-like epithelium (RhCE) models, including EpiOcular™ (MatTeck, Ashland, MA, USA), SkinEthic™ HCE (SkinEthic, Lyon, France), MCTT HCE™ (Biosolution, Suwon-si, Korea), and Labcyte Cornea model (JTE, Okinawa, Japan), have also been reported as in vitro alternative tests to eliminate species difference [12,13,14,15,16], presenting a high similarity of morphology, histology, and biomarker expression to human corneal epithelium [15].

Despite the importance as a criterion for eye irritation potential assessment in the RhCE model, assessment methods that rely solely on tissue viability still have limitations of low specificity associated with the high false-positive rate depending on the characteristics of the materials, especially for moderate to weak eye irritants [14,17,18]. Therefore, to improve the specificity and accuracy of in vitro test performance, a new additional biomarker for eye irritation should be discovered to enhance the predictive capability for eye irritation before the viability test. There has been an increasing interest in gene expression data as an alternative approach for identifying irritation potentials. The SENS-IS test protocol for the in vitro detection of sensitizers based on a 3D reconstructed human skin model (Episkin, France) was also introduced to quantitatively measure the expressions of 62 gene biomarkers for skin irritation and inflammation [19]. Moreover, the expression of heat shock protein 27 in human skin and a reconstructed organotypic skin model was proposed as a biomarker of an in vivo alternative test that reveals chemicals that can potentially cause skin irritation [20]. As treatment with skin sensitizer and irritant increased *IL-1α* and *IL-8* mRNA, they can be used as in vitro potential biomarkers that can test skin irritants in the reconstructed human epidermis [21]. Further, Braunstein et al. [22] proposed heme oxygenase-1 as a cell stress biomarker for ocular irritants in human corneal epithelial cell lines. Along with the identifications of several biomarkers to improve the predictive capability for local irritation, Choi et al. [23] previously identified that cornifelin and early growth response-1 (*Egr1*) expressions were upregulated in a RhCE model MCTT HCE treated with eye irritants by performing whole-gene microarray, suggesting their potential to serve as biomarkers for ocular toxicity.

In this study, our primary aim was to elucidate the change in EGR1 expression during ocular irritation and its potential mechanism using a variety of test materials in in vivo animal model and in vitro human corneal epithelial cell line model. Additionally, we further analyzed organ-specific features of EGR1 expression change related to the progression of multiorgan toxicity following the treatment of a well-known hepato- and nephrotoxicant in vivo to identify whether EGR1 expression can be regulated in a cell type-specific manner. The secondary aim of this study was to develop an *Egr1* promoter–luciferase gene reporter assay for an in vitro alternative method to predict in vivo cytotoxic potential in the processes of drug discovery and development.

## 2. Materials and Methods

### 2.1. Animal Experiment

Male BALB/c mice (6 weeks old, 20–22 g) and Sprague–Dawley rats (8 weeks old, 240–260 g) were purchased from Orient Bio (Sungnam, Korea) and raised in a specific-pathogen-free facility under normal atmospheric temperature (22 ± 2 °C) and relative humidity (55 ± 5%) for a 12 h light-dark cycle. Animals were fed a natural-ingredient diet Harlan 2918C (Raon Bio, Yongin, Korea) and tap water ad libitum. All animals were used for research after 3 days of acclimatization, and all experimental procedures were performed after approval by the Institutional Animal Care and Use Committee at The Catholic University of Korea. The first experiment using mice was divided into two groups, 1% sodium lauryl sulfate (SLS) and vehicle (phosphate-buffered saline, PBS) control (*n* = 3 per group) to characterize the histopathological changes during the ocular irritation process. The dose and method were adopted from the methods of Lin et al. [24] and Pauloin et al. [25] with minor modifications. The mice were treated with test material twice a day for 3 days at 12 h intervals. The mice were gently grasped, and 5 µL of each solution was treated to the right inferior conjunctival sac, and the eyes were kept open for 60 s. At 12 h after the last treatment, mice were anesthetized with isoflurane and sacrificed, and the eyes were fixed in Davidson’s solution for histopathological analysis. In the second experiment using rats, cisplatin was treated to induce hepatotoxicity and nephrotoxicity according to the method of Hwang et al. [26] and Hwang et al. [27]. Briefly, the experimental group was divided into two groups: normal saline and cisplatin treatment (*n* = 5 per group). Cisplatin was injected intraperitoneally at a single dose of 25 mg/kg. After each rat was anesthetized with isoflurane at 48 h following cisplatin injection, blood samples were collected from the postcaval vein and stored in vacutainer serum-separating tubes (BD Diagnostic, Sparks, MD, USA). The left lobe of the liver and kidney tissues were excised and immediately cryopreserved in liquid nitrogen for RNA and protein analysis.

### 2.2. Serum Collection and Biochemical Analysis

The collected blood samples were centrifuged at 3000 rpm for 15 min to separate serum. Serum was used to measure alanine aminotransferase (ALT), blood urea nitrogen (BUN), and creatinine. These biochemical parameters were measured using an automated clinical chemistry analyzer NX500 (Fujifilm, Tokyo, Japan).

### 2.3. Histology and Immunohistochemistry

Paraffin blocks were made using fixed mouse eyes and sectioned into 4 µm thickness. For histopathological analysis, hematoxylin and eosin staining were performed and observed through a light microscope (Leica, Hamburg, Germany). For immunohistochemistry, paraffin sections were deparaffinized with xylene, rehydrated with ethanol, and antigen retrieval was performed using sodium citrate buffer at 80 °C for 10 min. Peroxidase activity was blocked with 3% H_2_O_2_ and incubated overnight at 4 °C in primary EGR1 antibody (1:50 dilute in Dako antibody diluent, Cell signaling, Danvers, MA, USA). The secondary antibody and DAB detection reaction were performed using the Dako REAL™ EnVision™ Detection System kit (Dako, Glostrup, Denmark). Hematoxylin was used for counterstaining. The stained target was observed using a light microscope (Leica).

### 2.4. Cell Culture and Treatments

Immortalized human corneal epithelial cells and WB-F344 rat liver epithelial cells were obtained from Chung-Ang University (Seoul, Korea) and Seoul National University Hospital (Seoul, Korea), respectively. Human corneal epithelial cells were grown in EpiLife™ medium (Gibco, Grand Island, NY, USA) supplemented with 5% Human Corneal Growth Supplement (Thermo Fisher Scientific, Waltham, MA, USA) and 0.5% Penicillin-Streptomycin (Gibco) at 37 °C in a 5% CO_2_ incubator. WB-F344 cells were maintained in Dulbecco’s modified eagle’s medium F-12 (Gibco) with 10% fetal bovine serum (WelGENE, Daegu, Korea) and 1% antibiotic–antimycotic (Gibco). According to the methods of Ye et al. [28], we treated human corneal epithelial cells with SLS and benzalkonium chloride (BAC) as well as 17 test substances from various chemical classes (6 no category, 3 Cat2B, 2 Cat2A, and 6 Cat1) according to the UN GHS category of irritants [29] (Table 1). After irritant treatments for 10 min and washing with PBS, post-incubation was performed for 6, 12, or 18 h. Cisplatin was treated for 24 h according to the method of Hwang et al. [26]. Subsequently, RNA or protein was extracted for further experiments. For mitogen-activated protein kinase (MAPK) inhibitor treatment test, 50 µM extracellular signal-regulated kinase (ERK) inhibitor PD98059 (Sigma, St. Louis, MO, USA), 30 µM c-Jun N-terminal kinases (JNK) inhibitor SP600125 (Merck Millipore, Guyancourt, France), and 10 µM p38 inhibitor SB203580 (Selleck Chemicals, Houston, TX, USA) were added at 1 h at 37 °C before chemical treatment.

### 2.5. Cell Viability Assay

The viability of human corneal epithelial cells was measured using a CCK-8 assay kit (Dojindo, Kumamoto, Japan). Cells (at a density of 1–2 × 10^4^ cells) were seeded in 96-well plates and incubated for 48 h. Thereafter, the chemicals were treated with various concentrations for 10 min, washed with PBS, and grown in fresh medium for 6 h at 37 °C under 5% CO_2_. Subsequently, 10 µL CCK8 reagent was added to each well and reacted at 37 °C for 2 h, and the absorbance was measured at 450 nm using a SpectraMax 190 (Molecular Devices, LLC, Sunnyvale, CA, USA).

### 2.6. RNA Extraction, cDNA Synthesis, and Quantitative Real-Time Polymerase Chain Reaction (qPCR)

Total RNA was extracted using Trizol reagent (Invitrogen, Carlsbad, CA, USA), and its quantity and purity were determined by Nanodrop2000 (Thermo Fisher Scientific). Reverse transcription was carried out to synthesize cDNA using ReverTra Ace^®^ qPCR RT Master Mix (TOYOBO, Osaka, Japan); qPCR was performed using 1 µL cDNA with THUNDERBIRD^®^ SYBR^®^ qPCR Mix (TOYOBO) and the CFX connect™ Real-time PCR detection system (Bio-Rad, Hercules, CA, USA). The relative expression levels were calculated using the Cq (2^−ΔΔCq^) method [ΔCq = Cq (target gene) − Cq (housekeeping gene)]. TATA-binding protein (TBP) and glyceraldehyde 3-phosphate dehydrogenase (GAPDH) were used to normalize the relative expression levels. The primer sequences used for real-time PCR were as follows: human *Egr1* (forward: 5′-GACCGCAGAGTCTTTTCCTG-3′, reverse: 5′-AGCGGCCAGTATAGGTGATG-3′), human *TBP* (forward: 5′-ATAAGAGAGCCACGAACCAC-3′, reverse: 5′-CTCCTGTGCACACCATTTTC-3′), rat *Egr1* (forward: 5′-CCAGTGCCCACCTCTTACTC-3′, reverse: 5′-TGCAGACTGGAAGGTGCTG-3′), and rat *GAPDH* (forward: 5′-ATCACCATCTTCCAGGAGCGA-3′, reverse: 5′-CCTTCTCCATGGTGGTGAAGAC-3′).

### 2.7. Droplet Digital PCR (ddPCR)

ddPCR was performed to identify changes in the mRNA expressions of *Egr1*, *elongation of very long chain fatty acids protein 1* (*ELOVL1*), and *ELOVL4* by 17 chemicals from various chemical classes (6 no category, 3 Cat2B, 2 Cat2A, and 6 Cat1) according to the UN GHS category of irritants [29] (Table 1). The concentrations of the test substances were selected based on solubility and cell viability (>50%). QX200 Droplet Digital PCR System (Bio-Rad) consists of a C1000 Touch Thermal cycler, QX200 Droplet Generator, and QX200 Droplet Reader. The ddPCR mixture (20 µL) was made by mixing 10 µL of ddPCR supermix for EvaGreen (Bio-Rad), 1 µL of each primer (10 pmol), and 8 µL of DNA in distilled water extracted from human corneal epithelial cells. Droplets were generated by reacting the ddPCR mixture with the droplet generation oil (70 µL) for 2 min using a droplet generator. PCR conditions were initially started at 95 °C for 10 min, then 40 cycles were repeated at 94 °C for 30 s and 60 °C for 1 min. Droplet reading was performed using Quantasoft software (Bio-Rad). The primer sequences used for ddPCR were as follows: human *ELOVL1* (forward: 5′-TCTCCAAGTTCATTGAGCTGAT-3′, reverse: 5′-AGAGTGATGGAAGACATGTAGG-3′) and human *ELOVL4* (forward: 5′-GGCCCATGGATTCAGAAATA-3′, reverse: 5′-GGAAGGGGCAGTCAGTGTAA-3′). The *Egr1* primer set used the same as qPCR.

### 2.8. Western Blot

Cells were lysed with an appropriate amount of RIPA buffer (25 mM Tris-HCl pH 7.6, 150 mM NaCl, 0.1% SDS, 1% sodium deoxycholate, 1% Triton X-100) containing protease (Thermo Fisher Scientific) and phosphatase inhibitors (Thermo Fisher Scientific). The cell lysates were then collected and homogenized. After centrifuging at 15,000 rpm for 30 min at 4 °C, the supernatants were collected. Protein concentrations were measured using the Bradford assay (Bio-Rad). Sample proteins were subjected to polyacrylamide gel electrophoresis and transferred to nitrocellulose membranes (Bio-Rad). The membranes were then blocked with 5% skim milk in PBS with 0.1% Tween 20 for 2 h at room temperature and incubated overnight at 4 °C with primary antibodies against EGR1 (rabbit monoclonal IgG, 1:1000, Cell signaling), ERK1/2 (mouse monoclonal IgG, 1:1000, Santa Cruz, Santa Cruz, CA, USA), phospho-ERK1/2 (rabbit polyclonal IgG, 1:2000, Cell signaling), JNK (mouse monoclonal IgG, 1:1000, Santa Cruz), phospho-JNK (rabbit monoclonal IgG, 1:2000, Cell signaling), p38 (mouse monoclonal IgG, 1:1000, Santa Cruz), phospho-p38 (rabbit monoclonal IgG, 1:2000, Cell signaling), phospho-Akt (rabbit polyclonal IgG, 1:1000, Cell signaling), dual specificity phosphatase 2 (DUSP2; rabbit polyclonal IgG, 1:1000, Abcam), and β-actin (mouse monoclonal IgG, 1:5000, Santa Cruz). Following incubation with the horseradish peroxidase-conjugated secondary antibodies (Bio-Rad) for 1 h at room temperature, the protein bands were detected using an ECL kit (Bio-Rad). Protein bands were observed using ChemiDoc™ XRS+ System (Bio-Rad).

### 2.9. Putative Transcription Factor Binding Site in the Egr1 Promoter

The gene database for the proximal promoter region of *Egr1* was searched using the NCBI Entrez system (http://www.ncbi.nlm.nih.gov/gene, accessed on 27 April 2021). Potential transcription factor binding sites of the rat *Egr1* gene promoter region were identified (core binding site motif-match score, >90%) using the MATCH program (http://www.gene-regulation.com, accessed on 30 June 2021).

### 2.10. Egr1-Small Interfering RNA (siRNA) Silencing

*Egr1* siRNA (s4537; Invitrogen) was transfected into human corneal epithelial cells using Lipofectamine™ RNAiMAX (Invitrogen) according to the manufacturer’s protocol. Briefly, cells were seeded in plates (6 well, 1.5 × 10^5^ cells/well; 96 well, 1 × 10^4^ cells/well). After 48 h, siRNA (6 well, 40 pmol; 96 well, 1.6 pmol) and Lipofectamine™ RNAiMAX (6 well, 2 μL; 96 well, 0.08 μL) were diluted in Opti-MEM medium (Gibco) and mixed. The mixture was incubated at room temperature for 30 min and added to the cell. At 24 h after transfection, chemicals were treated for further analysis.

### 2.11. Egr1 Promoter–Luciferase Gene Reporter Assay

Changes in promoter activity responsible for the expression of the *Egr1* gene were assessed by amplifying fragments of the 5′-promoter region of the rat *Egr1* gene by PCR using forward primer (5′-CGACGCGTGTCCTTCCATATTAGGGCTTCC-3′) and reverse primer (5′-CCGCTCGAGCAGCATCATCTCCTCCAGTTT-3′) containing Mlu1 and Xho1 restriction enzyme sites, respectively. PCR was carried out using Ex-Taq DNA Polymerase (Takara, Tokyo, Japan). The PCR conditions were as follows: Initial denaturation at 95 °C for 5 min; 38 cycles of 95 °C for 30 s, 62 °C for 30 s, 72 °C for 1 min; and final extension at 72 °C for 5 min. The PCR products were purified with Wizard^®^ SV Gel and PCR Clean-Up System (Promega, Madison, WI, USA), digested with Mlu1 and Xho1, and cloned into a pGL3-basic vector. The digested products were ligated using T4 DNA Ligase (Takara). The pGL3 vectors ligated with the PCR products were transformed into competent *E. coli* DH5α cells by the heat shock method. Transformed DH5α cells were spread on an LB agar plate containing ampicillin, and the colonies were confirmed. A colony containing the *Egr1* promoter-reporter vector was selected, cultured in LB broth, and the plasmid was isolated using the Qiagen Miniprep kit (Qiagen, Valencia, CA, USA). WB-F344 cells were seeded in a 48-well plate and transfected with *Egr1* promoter-reporter vector for 24 h using lipofectamine 3000 (Invitrogen). The dual-luciferase reporter assay kit (Promega) was used for the assay according to the manufacturer’s instructions. In this system, firefly luciferase was used as the experimental promoter-reporter and renilla luciferase as the control reporter. The luciferase activity was measured by adding luciferin, which is the substrate of luciferase to the lysates. Luminescence was then measured using a GloMax^®^ Explorer (Promega). Luciferase activity was calculated from the ratio of the firefly and renilla luciferase activity.

### 2.12. Statistics

All results were expressed as means ± standard deviation (SD). Comparisons between two or more groups were analyzed by student’s *t*-test or one-way analysis of variance using SPSS version 19 software (SPSS Inc., Chicago, IL, USA). Values indicating statistical significance were set at *p* < 0.05. The data from the analyses were expressed using GraphPad Prism 8 software (GraphPad Software, San Diego, CA, USA).

## 3. Results

### 3.1. Egr1 Expression Alterations Associated with Multiorgan Injury In Vivo

**Figure 1:**
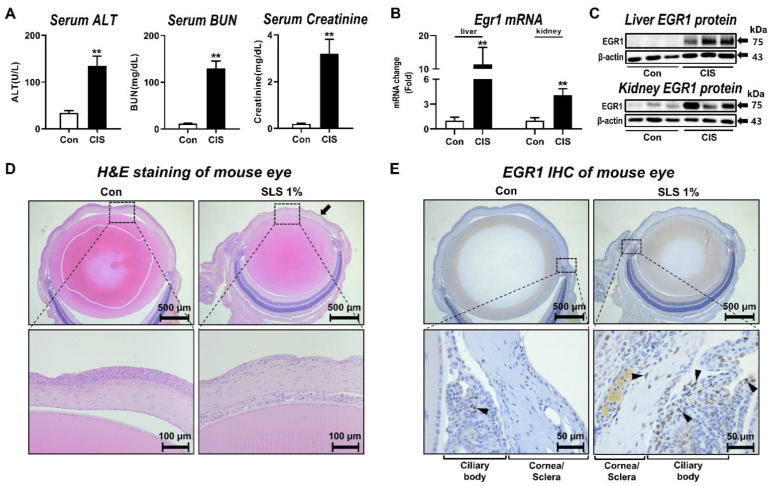
Alteration of EGR1 expression by hepatotoxicity, nephrotoxicity, and ocular irritation. (**A**) Changes in liver and kidney injury biomarkers after cisplatin treatment to rats for 48 h (*n* = 5 per group). (**B**) Inductions of hepatic and renal Egr1 mRNA expressions at 48 h after cisplatin treatment to rats. (**C**) Increased expressions of hepatic and renal EGR1 protein at 48 h after cisplatin treatment in rats. (**D**) Representative hematoxylin and eosin staining showing cornea epithelium elimination (closed arrows) in SLS-treated mouse eye. (**E**) Immunostaining of EGR1 protein (arrowheads) in SLS-treated mouse eye. Data are expressed as mean ± SD (** *p* < 0.01). Con, control; CIS, cisplatin; SLS, sodium lauryl sulfate; H&E, hematoxylin and eosin; IHC, immunohistochemistry.

To demonstrate the change in EGR1 expression under situations of multiorgan injury, we first injected the rats with cisplatin, which has been known to induce nephrotoxicity and hepatotoxicity as major side effects [26,30]. Serum liver injury biomarker ALT and kidney injury biomarkers BUN and creatinine [31,32] were significantly elevated in rats at 48 h after the administration of cisplatin (Figure 1A). In comparison with the control group, EGR1 mRNA and protein levels in the liver were significantly increased after the cisplatin exposure (Figure 1B). Similarly, cisplatin led to significant induction of the expressions of kidney EGR1 mRNA and protein in the presence of cisplatin-induced nephrotoxicity (Figure 1C).

To further determine the change in EGR1 expression during ocular irritation in vivo, a well-known eye irritant SLS [23] was applied to the mouse eye. Histological examination revealed that most of the cornea epithelium was eliminated in the SLS-treated group (Figure 1D). Notably, there was strong immunohistochemical staining of nuclear EGR1 protein [33] in the eyes, especially in and around the ciliary body, of SLS-treated animals (Figure 1E).

### 3.2. In Vitro Egr1 Expression Alterations in Human Corneal Epithelial Cells by Treatment with Various Irritants

**Figure 2:**
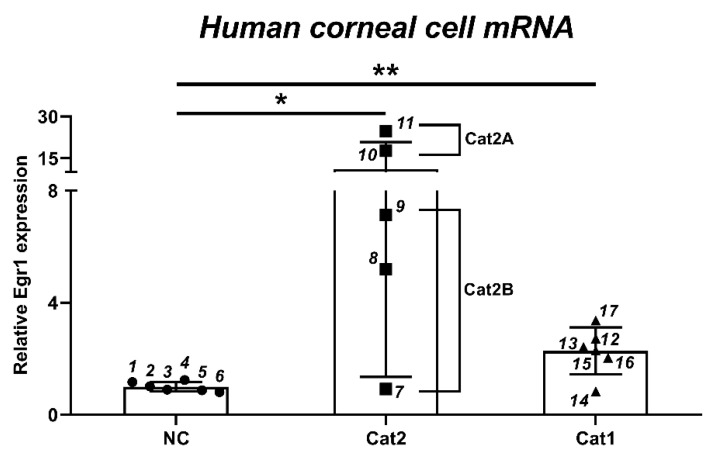
Changes of *Egr1* mRNA expression in human corneal epithelial cells by 17 test substances from various chemical classes. Human corneal epithelial cells were treated with test chemical solution for 10 min and incubated for 6 h. Each data was measured by ddPCR. Data are expressed as mean ± SD (* *p* < 0.05, ** *p* < 0.01). 1: 2-ethoxyethyl methacrylate; 2: piperonyl butoxide; 3: 1-ethyl-3-methylimidazolium ethylsulfate; 4: potassium tetrafluoroborate; 5: polyoxyl 40 hydrogenated castor oil; 6: dipropyl disulfide; 7: 2-methyl-1-pentanol; 8: diethyl tolumide; 9: 1,4-dibutoxy benzene; 10: 2,4,11,13-tetraazatetradecane diimidamide, N,N″-bis(4chlorophenyl)-3,12-diimino-, di-D-gluconate; 11: gamma-butyrolactone; 12: (ethylenediamine-propyl)-trimethoxysilane; 13: tetraethylene glycol diacrylate; 14: 1,2-benzisothiazol-3(2H)-one; 15: 3,5-dimethyl-2,5-hexanediol; 16: disodium 2,2′-([1,1′-biphenyl]-4,4′diyldivinylene)bis-(benzenesulfonate); 17: sodium oxalate. Cat1, serious damage; Cat2A, irritant; Cat2B, mild irritant; NC, no damage.

To clarify whether a variety of eye irritants can induce *Egr1* expression in vitro, we analyzed *Egr1* mRNA expressions after treatment with 17 test substances from the various classification of eye irritation (6 no category, 3 Cat2B, 2 Cat2A, and 6 Cat1) (Table 1) in human corneal epithelial cells. Based on the results of ddPCR, *Egr1* mRNA expressions were upregulated by two out of three Cat2B irritants (0.92-fold for #7 irritant; 5.19-fold for #8 irritant; 7.14-fold for #9 irritant) at a concentration showing more than 50% cell viability (Figure 2). Additionally, *Egr1* expression was strongly enhanced by 2 Cat2A irritants (17.67-fold for #10 irritant; 24.66-fold for #11 irritant). Five out of six Cat1 irritants significantly increased *Egr1* mRNA expression (2.72-fold for #12 irritant; 2.43-fold for #13 irritant; 0.84-fold for #14 irritant; 2.29-fold for #15 irritant; 2.04-fold for #16 irritant; 3.37-fold for #17 irritant).

**Figure 3:**
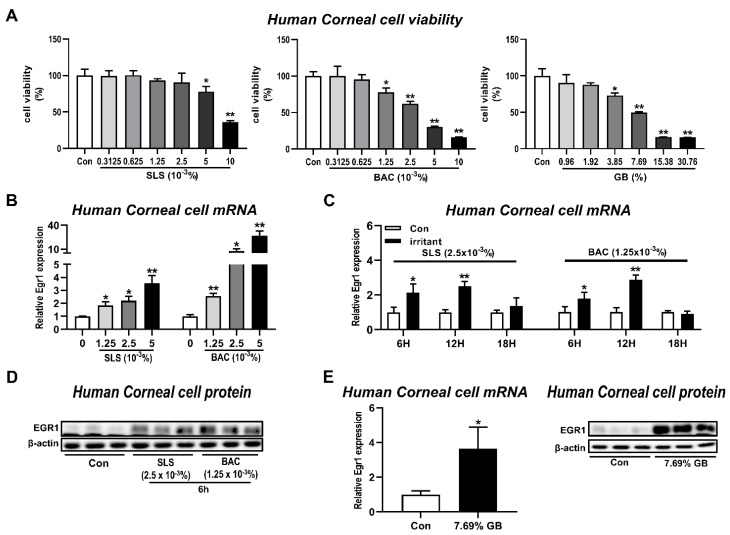
Induction of EGR1 expression in human corneal epithelial cells by irritant treatments. Human corneal epithelial cells were treated with various concentrations of test chemical solution for 10 min and incubated for 6, 12, and 24 h. (**A**) Effects of SLS, BAC, and GB on cell viability in human corneal epithelial cells post-incubation for 6 h. (**B**) Concentration-dependent increases in *Egr1* mRNA levels following SLS and BAC treatment and post-incubation for 6 h. (**C**) Changes in *Egr1* mRNA expressions according to post-incubation time (6, 12, and 18 h) after SLS and BAC treatment. (**D**) Changes in EGR1 protein levels induced by SLS and BAC. (**E**) Inductions of EGR1 mRNA and protein in GB-treated human corneal epithelial cells post-incubation for 6 h. TBP and β-actin were used as loading controls for mRNA analysis and protein-expression analysis, respectively. Data are expressed as mean ± SD (* *p* < 0.05 and ** *p *< 0.01). Con, control; SLS, sodium lauryl sulfate; BAC, benzalkonium chloride; GB, gamma-butyrolactone.

The irritant-induced increase of *Egr1* mRNA expression was confirmed by treating human corneal epithelial cells with SLS and BAC, representative anionic and cationic surfactants [23], as positive controls for irritation. In the CCK assay performed to measure cell viability at different concentrations of SLS, BAC, and gamma-butyrolactone (GB), we found the dose-dependent cytotoxicity after treatments of SLS (5 × 10^−3^% and 1 × 10^−2^%), BAC (1.25 × 10^−3^%, 2.5 × 10^−3^%, 5 × 10^−3^%, and 1 × 10^−2^%), and GB (3.85%, 7.69%, 15.38%, and 30.76%; Figure 3A). *Egr1* mRNA levels were increased by 1.85-, 2.2-, and 3.55-fold after SLS treatments at concentrations of 1.25 × 10^−3^%, 2.5 × 10^−3^%, and 5.0 × 10^−3^%, respectively, and post-incubation period for 6 h (Figure 3B). Similarly, BAC treatment also caused the dose-dependent elevations of *Egr1* mRNA expressions by 2.56-, 7.5-, and 27.17-fold at concentrations of 1.25 × 10^−3^%, 2.5 × 10^−3^%, and 5.0 × 10^−3^%, respectively. After SLS treatment (2.5 × 10^−3^%), *Egr1* mRNA expressions were elevated post-incubation for 6 h (2.15-fold) and 12 h (2.51-fold), not 18 h, compared with their corresponding control groups (Figure 3C). Further, BAC treatment upregulated *Egr1* mRNA expression by up to 1.77-fold and 2.87-fold post-incubation for 6 h and 12 h, respectively. Consistent with the increased mRNA levels, EGR1 protein expressions were also increased in both SLS and BAC groups (Figure 3D). In the case of GB in category Cat2A selected based on the results of ddPCR (Figure 2), we found a significant increase in the expression of *Egr1* mRNA by 3.64-fold (Figure 3E). Likewise, GB significantly increased the protein expression of EGR1.

### 3.3. Involvement of ERK Pathway in the Elevation of EGR1 Expression after Irritation Exposure

**Figure 4:**
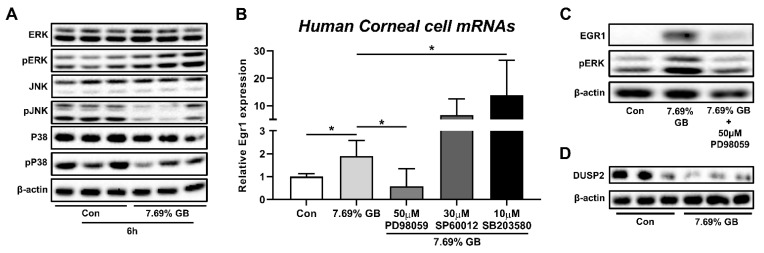
Involvement of MAPK signaling pathway in EGR1 upregulation after exposure to GB. Human corneal epithelial cells were treated with GB for 10 min and incubated for 6 h. For MAPK inhibitor treatment test, ERK inhibitor PD98059, JNK inhibitor SP600125, and p38 inhibitor SB203580 were added 1 h before chemical treatment. (**A**) Effects of GB treatment on phosphorylation of ERK, JNK, and p38. (**B**) Effects of ERK inhibitor (PD98059), JNK inhibitor (SP60012), and p38 inhibitor (SB203580) on GB-induced increase in *Egr1* mRNA expression. (**C**) Effect of ERK inhibitor (PD98059) on GB-induced increase in EGR1 protein expression. (**D**) Reduction of DUSP2 protein expression after GB treatment. TBP and β-actin were used as loading controls for mRNA analysis and protein-expression analysis, respectively. Data are expressed as mean ± SD (* *p* < 0.05). Con, control; GB, gamma-butyrolactone.

As EGR1 expression has been known to be regulated by the MAPK/ERK pathway [34,35], a further experiment was performed to determine the involvement of MAPK signaling pathways in EGR1 expression upregulated by irritants in human corneal epithelial cells. At first, ERK phosphorylation was increased by treatment with GB 7.69%, whereas phosphorylation of JNK and p38 was decreased (Figure 4A). Thereafter, this observation was further confirmed by experiments using inhibitors of the ERK pathway (PD98059), JNK pathway (SP60012), and p38 pathway (SB203580; Figure 4B). GB-mediated increases in *Egr1* mRNA expressions were significantly promoted by the pretreatment of 10 μM p38 inhibitor SB203580. Furthermore, markedly elevated levels of *Egr1* mRNA in GB-exposed cells showed aggravating trends in 30 μM JNK inhibitor SP60012 pretreatment cells (*p* = 0.123). In contrast, pretreatment of 50 μM ERK inhibitor PD98059 significantly suppressed GB-induced elevation of *Egr1* mRNA expression. Consistently, ERK inhibitor PD98059 also blocked GB-induced EGR1 protein elevation (Figure 4C), confirming the potential involvement of cross-talk between the MAPK signaling pathways, including ERK, JNK, and p38, in EGR1 expression regulation. In addition, the protein expression of DUSP2, which is produced by the JNK signaling pathway and inhibits the ERK pathway by dephosphorylating pERK [36,37,38], was reduced by GB treatment in human corneal epithelial cells (Figure 4D).

### 3.4. Effects of Egr1 Knockdown in Human Corneal Epithelial Cells Treated with Irritants

**Figure 5:**
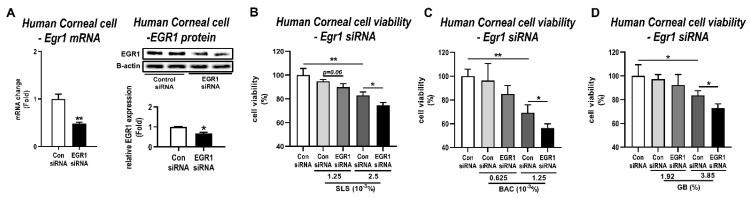
Effects of siRNA-mediated *Egr1* knockdown in irritant-induced cytotoxicity. At 24 h after transfection of *Egr1* siRNA into human corneal epithelial cells, the cells were treated with various concentrations of SLS, BAC, and GB for 10 min and incubated for 6 h. (**A**) Knockdown of EGR1 mRNA and protein by *Egr1* siRNA transfection. (**B**–**D**) Effects of *Egr1* knockdown in cytotoxicity induced by SLS (**B**), BAC (**C**), and GB (**D**). TBP and β-actin were used as loading controls for mRNA analysis and protein-expression analysis, respectively. Data are expressed as mean ± SD (* *p* < 0.05, ** *p* < 0.01). Con, control; SLS, sodium lauryl sulfate; BAC, benzalkonium chloride; GB, gamma-butyrolactone.

EGR1 can play an essential role in promoting regeneration and proliferation under situations of stress and injury [39,40,41]. Thus, to identify the main reason for EGR1 upregulation after irritant exposure, we used siRNA to target the *Egr1* gene and investigated its effect in human corneal epithelial cells treated with irritants. At first, the knockdown efficiency of *Egr1* siRNA was confirmed by qPCR and Western blot. As expected, Egr1 siRNA significantly suppressed the EGR1 mRNA and protein levels in human corneal epithelial cells by 52% (*p* < 0.01) and 32% (*p* < 0.05), respectively (Figure 5A). Based on the result of the cell viability (Figure 3A), we selected the optimal concentrations of these irritants for *Egr1* siRNA assay (SLS: 1.25 × 10^−3^% and 2.5 × 10^−3^%; BAC: 0.625 × 10^−3^% and 1.25 × 10^−3^%; GB: 1.92% and 3.85%). As a result, pretreatment of Egr1 siRNA aggravated SLS-induced cytotoxicity at concentrations of 1.25 × 10^−3^% (94.76% live cells for Con siRNA vs. 89.88% for *Egr1* siRNA, *p* = 0.06) and 2.5 × 10^−3^% (82.92% liver cells for Con siRNA vs. 74.46% for *Egr1* siRNA, *p* < 0.05; Figure 5B). Knockdown of *Egr1* by siRNA markedly decreased cell viability in the group treated with 1.25 × 10^−3^% BAC (69.32% liver cells for Con siRNA vs. 56.38% for *Egr1* siRNA, *p* < 0.05; Figure 5C). Similarly, siRNA-medicated *Egr1* knockdown significantly promoted cytotoxicity induced by 3.85% GB compared to the corresponding control group (83.59% live cells for Con siRNA vs. 73.08% for Egr1 siRNA, *p* < 0.05; Figure 5D). Interestingly, in the low-dose group for BAC (0.625 × 10^−3^%) and GB (1.92%) showing almost normal viability, siRNA did not influence the cytotoxicity. These results indicate that the Egr1 knockdown by siRNA increases the susceptibility of eye irritation; therefore, the change in EGR1 caused by the irritants is likely to be a compensatory upregulation for the tissue repair process.

### 3.5. Induction of Egr1 Promoter Activity by Various Irritants

**Figure 6:**
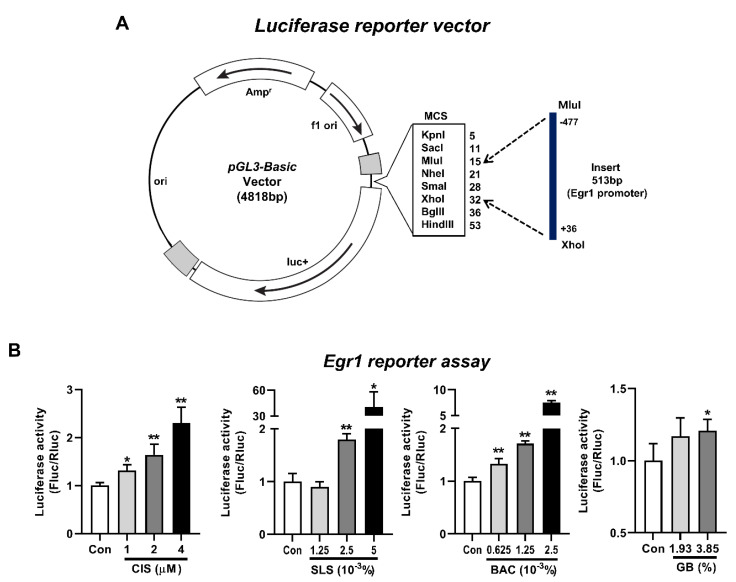
Induction of *Egr1* promoter activity by various irritants. At 24 h after transfection of *Egr1* promoter-reporter vector into WB-F344 cells, the cells were treated with various concentrations of cisplatin for 24 h. For eye irritants SLS, BAC, and GB, the cells were treated for 10 min and incubated for 6 h. (**A**) Structure of a luciferase reporter vector containing the *Egr1* gene promoter between the positions −477 and +36. (**B**) Inductions of *Egr1* promoter activity following the treatments of cisplatin, SLS, BAC, and GB. Data are expressed as mean ± SD (* *p* < 0.05 and ** *p* < 0.01). Con, control; CIS, cisplatin; SLS, sodium lauryl sulfate; BAC, benzalkonium chloride; GB, gamma-butyrolactone.

To further propose EGR1 as a potential biomarker for a high-throughput in vitro eye irritation test, induction of *Egr1* promoter activity in irritant-treated cells was analyzed by a luciferase reporter assay. After a luciferase reporter construct containing the *Egr1* gene proximal promoter between the positions −477 and +36 (Figure 6A) was transiently transfected into the WB-F344 cells, we treated the transfected WB-F344 cells with four representative toxicants and irritants. Along with the dose-dependent increases in *Egr1* mRNA expression level by cisplatin treatment in WB-F344 cells (Figure S1), cisplatin treatment led to significant inductions of *Egr1* promoter activity (1.31-fold for 1 µM; 1.64-fold for 2 µM; 2.3-fold for 4 µM) in a concentration-dependent manner (Figure 6B). In addition, *Egr1* promoter activity was significantly elevated in all of the groups treated with SLS (1.8-fold for 2.5 × 10^−3^%; 40.69-fold for 5 × 10^−3^%), BAC (1.33-fold for 0.625 × 10^−3^%; 1.71-fold for 1.25 × 10^−3^%; 7.52-fold for 2.5 × 10^−3^%), and GB (1.21-fold for 3.85%).

## 4. Discussion

Although various 3D RhCE models are widely used as in vitro alternative tests for eye irritation, these methods are critically dependent on tissue viability for distinguishing irritants from non-irritants [12,13,14,15,16], which has several limitations, including low specificity and a high false-positive rate of test results [14,17,18]. In this situation, gene expression screening for ocular irritation potential can enhance the prediction rate of a prediction model based on cell viability [23]. To discover a new gene biomarker to improve the accuracy of in vitro predictions for ocular irritation in the processes of drug discovery and development, we focused on *Egr1* gene expression, which has previously been known to be upregulated by eye irritants through whole-gene microarray [23]. Along with the understanding of the molecular mechanisms of the eye irritant-mediated EGR1 upregulation, we further identified whether EGR1 expression change related to the toxic response can be regulated in a cell type-specific manner in various organs such as the liver, kidney, and eye.

In the present study, significant increases in EGR1 mRNA and protein expressions were observed in liver and kidney tissues treated with cisplatin along with inductions of serum biochemical parameters associated with hepatotoxicity and nephrotoxicity. Additionally, immunohistochemistry for EGR1 revealed that the expression of EGR1 protein was increased in SLS-induced mouse eye irritation with damaged cornea epithelium. As one of the zinc-finger family transcription factors involved in cell proliferation, differentiation, apoptosis, and development [42,43], Lai et al. [39] demonstrated that hepatic EGR1 expression was activated rapidly by partial hepatectomy. However, liver regeneration was delayed in mice lacking EGR1 with decreased expressions of Cyclin D1, Cyclin E, and proliferating cell nuclear antigen, indicating that EGR1 is an important factor for wound healing, regeneration, and proliferation in response to various types of toxicity and injury. In addition to the changes in EGR1 expression in response to various injuries in vivo, we confirmed the ocular irritants-induced *Egr1* upregulation in vitro immortalized human corneal epithelial cells treated with 17 test substances from non-irritant to severe irritants according to the UN GHS category of irritants. We also found that EGR1 mRNA and protein levels increased after treatment with representative irritants SLS and BAC [23] as well as GB (classified as Category 2A), which induced the greatest increase in *Egr1* mRNA expression in the present study.

EGR1 expression can be regulated via various pathways. Cabodi et al. [44] demonstrated that activation of the PI3K/Akt pathway plays an important role in regulating *Egr1* expression. However, we found no significant change of Akt phosphorylation following GB treatment in human corneal epithelial cells (Figure S2). Meanwhile, the MAPK pathway regulates various physiological processes, including cell growth, differentiation, and apoptotic cell death [45]. Among the three main protein kinase families of MAPK, namely the ERKs, JNKs, and p38 family [46], the activated ERK pathway has been known to regulate a multitude of cellular responses such as cell proliferation, cell differentiation, cell survival, and cell motility by phosphorylating various transcription factors [46,47]. The activated JNKs phosphorylate downstream substrates including c-Jun, Ets-like-1 (Elk-1), p53, and Smad3 [46]. Prolonged activation of the JNK pathway induced by stress generally results in apoptosis, whereas transient activation of the JNK pathway induced by growth factor promotes cell survival and proliferation [36,46]. In response to various environmental stresses including heat shock, osmotic and oxidative stresses, pro-inflammatory cytokines, and tumor necrosis factor receptor signaling, the activation of the p38 pathway is related to cell motility, transcription, and chromatin remodeling [46]. To reveal the molecular mechanisms of the eye irritant-mediated EGR1 upregulation, we determined the changes of three main protein kinase families of MAPK following the exposure to eye irritant GB inducing the EGR1 expressions. In the present study, we have elucidated that ERK phosphorylation was upregulated by GB treatment, whereas the JNK and p38 phosphorylation were downregulated. Moreover, GB-mediated increases in *Egr1* mRNA expressions were significantly prevented by inhibition of ERK, and it was promoted by inhibition of JNK and p38. In particular, we found that the expression of DUSP2 protein, which is produced by the JNK signaling pathway and acts as a known negative regulator of the ERK [36,37,38], was downregulated by GB treatment. Additionally, it has been known that the protein phosphatase 2A stimulated by p38 MAPK can inhibit the ERK MAPK pathway by dephosphorylating ERK [48,49,50]. Collectively, it is more likely that these support our potential hypothesis on the involvement of ERK, JNK, and p38 in EGR1 upregulation under situations of injury.

Deletion of EGR1 exacerbated liver fibrosis of long-term acetaminophen-induced hepatotoxicity in mice [41]. In addition, tendon injury-related healing and repair were impaired by reducing tendon-associated collagen in the EGR1-knockout mouse [40]. Our group previously used a pre-biopsy method, in which liver tissues were biopsied before administering test material, and RNA sequencing to uncover the key inherent genes associated with the difference of drug-induced hepatotoxicity susceptibility. Using this approach, we demonstrated that *Egr1* mRNA expression was inherently lower before drug exposure in cisplatin-sensitive animals [26]. With regards to this, we found that siRNA targeting *Egr1* significantly suppressed the EGR1 mRNA and protein levels in human corneal epithelial cells and aggravated SLS-, BAC-, and GB-induced cytotoxicity in human corneal epithelial cells in this study. In contrast, *Egr1* siRNA had little or no effect on cell viability in the low-dose group for SLS, BAC, and GB. Thus, these results may reflect that irritant-mediated changes of EGR1, which plays a role in promoting wound healing, regeneration, and cell proliferation via a mechanism involving the ERK signaling [39,40,41], can be a compensatory upregulation for tissue repair. More importantly, our findings suggest that low expression of EGR1 can contribute to severe toxicity, possibly through a delay of tissue injury-related recovery processes, rather than causing toxicity by itself.

Although the concentration-dependent increases in EGR1 mRNA and protein were found after various irritant treatments, qPCR and Western blot have several disadvantages. Various complex steps including RNA/protein extraction, electrophoresis, and relative comparison are required in comparison to rapid and accurate evaluation methods. The assay using cells transfected with a pGL3-basic vector containing the rat *Egr1* gene promoter region from −477 to +36 showed a dose-dependent increase in luciferase activity in the presence of SLS, BAC, GB, and cisplatin, demonstrating the high sensitivity of the *Egr1* reporter vector that can discriminate irritants. In contrast, the expression level of EGR1 remains to be determined as criteria for classifying irritation. In the present study, we found serum response factor (SRF), Elk-1, and Ets-1 as the top three transcription factors with the highest frequencies of binding sites in the proximal promoter region of *Egr1* (~500 bp) based on the results of bioinformatics analysis (Figure S3 and Table S1). When ERK is phosphorylated by the MAPK kinase, ERK is activated and translocated into the nucleus. Then the activated ERK phosphorylates several transcription factors including SRF, Elk-1, and Ets-1 [46,51,52,53] and bind to the promoter region of various genes including *Egr1*, which is a transcription factor associated with cell proliferation and differentiation [54] and induced by diverse stimuli such as growth factors, differentiation signals, and DNA-damaging agents such as UV light and ionizing radiation [55]. Future research should explore the transcriptional factor that binds to the *Egr1* proximal promoter for irritant-mediated transcription through the ERK signaling pathway.

In summary, we first found the upregulation of EGR1 associated with a delay of injury-related recovery via ERK signaling pathway under situations of toxic injury. More importantly, we produced a high-throughput luciferase assay capable of identifying in vivo cytotoxic potential using a reporter construct containing the *Egr1* gene promoter region. Remarkably, our findings elucidated a key role of EGR1 as a potential biomarker in improving the predictive capacity of in vitro eye irritation tests used for quantifying tissue viability in immortalized human corneal epithelial cells. Despite the high probability of this evaluation system, the *Egr1* promoter–luciferase gene reporter assay developed in this study should be verified to improve the specificity and sensitivity using various eye irritants according to the degree of irritation. In the current study, we also found that mRNA expression levels of *ELOVL1* and *ELOVL4*, which have been known to play an important role in maintaining the skin barrier function [56], were also significantly increased by treatment of various irritants (UN GHS Cat1 and Cat2) in human corneal epithelial cells (Figure S4). In particular, it is noteworthy that increases in *ELOVL1* and *ELOVL4* mRNA levels were identified even for Cat2B #7 irritant (Egr1, 0.92-fold vs. control; ELOVL1, 1.69-fold; ELOVL4, 1.66-fold) and Cat1 #14 irritant (Egr1, 0.84-fold vs. control; ELOVL1, 1.39-fold; ELOVL4, 1.39-fold), which did not cause *Egr1* upregulation (Figure 2 and Table 1). Thus, further experiments are needed to reduce the potential false negative of this *Egr1* reporter assay by combining more gene biomarkers, including ELOVL1 and ELOVL4. Moreover, the assay used in this study can be a valuable screening method that can detect multi-organ damage depending on the type of cells after additional research regarding the detailed mechanism on the relationship between EGR1 expression and multiorgan failure having diverse pathogenesis.

## Figures and Tables

**Table 1:** Characteristic and eye irritation information of 17 test substances for ddPCR analysis.

No.	Test Chemicals	CAS No.	Physical State	Supplier	Category ^a^	Conc. ^b^
1	2-Ethoxyethyl methacrylate	2370-63-0	liquid	Sigma-Aldrich	NC	10%
2	Piperonyl butoxide	1951-03-06	liquid	Toronto research chemicals, Toronto, Canada	NC	10%
3	1-Ethyl-3-methylimidazolium ethylsulfate	342573-75-5	liquid	Sigma-Aldrich	NC	10%
4	Potassium tetrafluoroborate	14075-53-7	solid	Sigma-Aldrich	NC	10%
5	Polyoxyl 40 hydrogenated castor oil	61788-85-0	viscous	Sigma-Aldrich	NC	10%
6	Dipropyl disulfide	629-19-6	liquid	Sigma-Aldrich	NC	10%
7	2-Methyl-1-pentanol	105-30-6	liquid	Sigma-Aldrich	Cat2B	1.25%
8	Diethyl tolumide	134-62-3	liquid	Tokyo Chemical Industry, Tokyo, Japan	Cat2B	1.92%
9	1,4-Dibutoxy benzene	104-36-9	solid	Tokyo Chemical Industry	Cat2B	5%
10	2,4,11,13-Tetraazatetradecane diimidamide, N,N″-bis(4chlorophenyl)-3,12-diimino-, di-d-gluconate	18472-51-0	liquid	Sigma-Aldrich	Cat2A	0.05%
11	Gamma-butyrolactone	96-48-0	liquid	Sigma-Aldrich	Cat2A	7.69%
12	(Ethylenediamine-propyl)-trimethoxysilane	1760-24-3	liquid	Sigma-Aldrich	Cat1	0.25%
13	Tetraethylene glycol diacrylate	17831-71-9	liquid	Sigma-Aldrich	Cat1	0.01%
14	1,2-Benzisothiazol-3(2*H*)-one	2634-33-5	solid	Sigma-Aldrich	Cat1	0.01%
15	3,5-Dimethyl-2,5-hexanediol	110-03-2	solid	Sigma-Aldrich	Cat1	0.1%
16	Disodium 2,2′-([1,1′-biphenyl]-4,4′diyldivinylene)bis-(benzenesulfonate)	27355-41-8	solid	Tokyo Chemical Industry	Cat1	1%
17	Sodium oxalate	62-76-0	solid	Sigma-Aldrich	Cat1	3%

^a^ GHS, Globally Harmonized System of Classification and Labeling of Chemicals; Cat1, serious damage; Cat2A, irritant; Cat2B, mild irritant; NC, no damage. ^b^ Chemical concentration in the experiment to confirm *Egr1* mRNA expression quantity through ddPCR.

## Data Availability

The data presented in this study are available from the corresponding author on reasonable request.

## Supplementary Materials

Figure S1. *Egr1* mRNA expression levels after cisplatin administration in WB-F344 cells, Figure S2. Effect of GB treatment on phosphorylation of Akt in human corneal epithelial cells, Figure S3. Promoter region of rat Egr1 and putative binding sites for three transcription factors SRF, Elk-1, and c-ETS-1, Figure S4. Changes of mRNA expressions of *ELOVL1* and *ELOVL4* in human corneal epithelial cells by 17 test substances from various chemical classes. Table S1. Putative transcription factor binding sites in *Egr1* promoter region between the positions −477 and +36 (Top 3).
