# Supplementary material for: Egr1 Gene Expression as a Potential Biomarker for In Vitro Prediction of Ocular Toxicity
Source: Pharmaceutics. 2021 Sep 29;13(10):1584. doi: 10.3390/pharmaceutics13101584 (PMC8537669; doi:10.3390/pharmaceutics13101584)
Supplement: Supplementary file 1 [file pharmaceutics-13-01584-s001.zip › pharmaceutics-1340407-supplementary.pdf]

pharmaceutics

Supplementary Materials: *Egr1* Gene Expression as a Potential Biomarker for In Vitro Prediction of Ocular Toxicity

Da-Bin Hwang, Shin-Young Kim, Dong-Hoon Won, Changuk Kim, Yoo-Sub Shin, Jong-Hwan Park, Young-Jin Chun, Kyung-Min Lim, Jun-Won Yun

Table S1. Putative transcription factor binding sites in *Egr1* promoter region between the positions −477 and +36 (Top 3).

Factor	Start position	End position	Strand	Core match	Matrix match	Sequence[a]
SRF	-473	-459	(+)	1.000	0.927	ttCCATAttagggc
	-453	-435	(-)	1.000	0.951	cttcccataTATGGccat
	-453	-438	(-)	1.000	0.979	cttcCCATAtatggc
	-451	-437	(+)	1.000	0.985	tcCCATAtatggcc
	-451	-437	(-)	1.000	0.987	tcccataTATGGcc
	-450	-435	(+)	1.000	0.991	cccataTATGGccat
Elk-1	-465	-449	(-)	1.000	0.888	tagggCTTCCtgcttc
	-463	-449	(-)	0.938	0.928	gggcTTCCTgcttc
	-310	-296	(-)	1.000	0.949	cagcTTCCGccgcc
c-ETS-1 (p54)	-462	-452	(-)	0.947	0.962	ggctTCCTGc
	-169	-159	(-)	1.000	0.981	aacaTCCGGc
	-94	-84	(-)	1.000	0.968	cacgTCCGGg

[a]Capital letters indicate the positions in the sequence which match with the core sequence of the matrix, while the lower cases refer to the remaining position of a matrix.

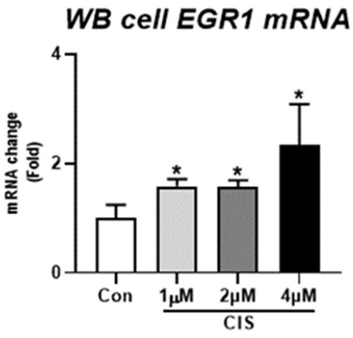

Figure S1. Egr1 mRNA expression levels after cisplatin administration in WB-F344 cells. Values are expressed as relative mRNA expression that has been normalized to GAPDH. Data expressed as means ± SD (**p < 0.01).

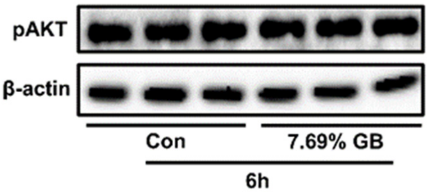

Figure S2. Effect of GB treatment on phosphorylation of Akt in human corneal epithelial cells. Human corneal epithelial cells were treated with GB for 10 min and incubated for 6 h. β-actin was used as loading control for protein-expression analysis.

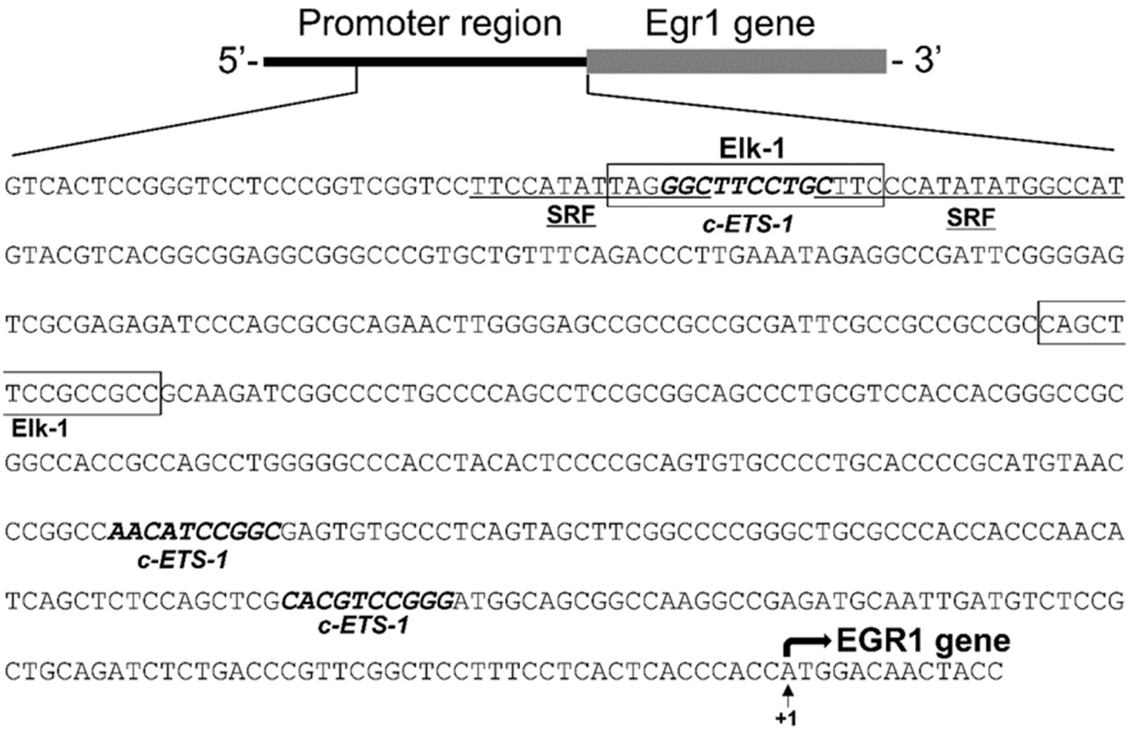

Figure S3. Promoter region of rat Egr1 and putative binding sites for three transcription factors SRF (underlined), Elk-1 (labeled box), and c-ETS-1 (bold and italic).

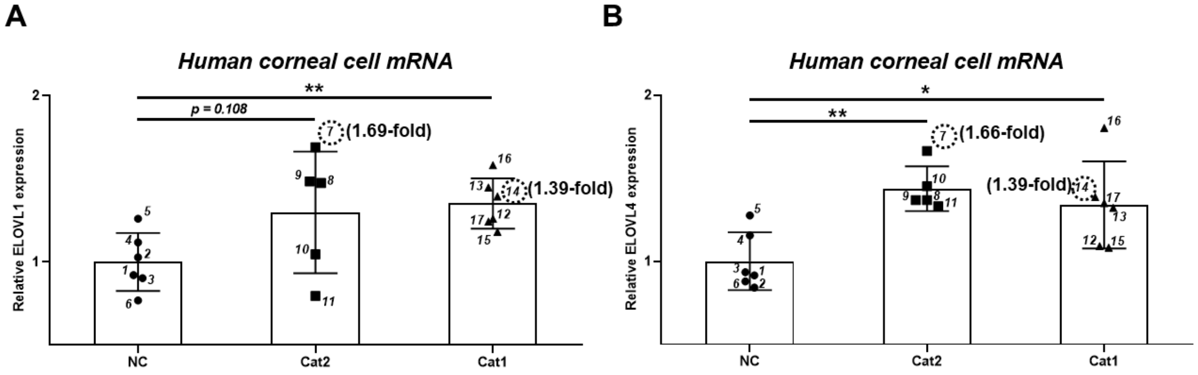

Figure S4. Changes of mRNA expressions of ELOVL1 and ELOVL4 in human corneal epithelial cells by 17 test substances from various chemical classes. Human corneal epithelial cells were treated with test chemical solution for 10 min and incubated for 6 h. Each data for ELOVL1 (A) and ELOVL4 (B) was measured by ddPCR. Data are expressed as mean ± SD (*$p < 0.05$, **$p < 0.01$). 1: 2-Ethoxyethyl methacrylate; 2: Piperonyl butoxide; 3: 1-Ethyl-3-methylimidazolium ethylsulphate; 4: Potassium tetrafluoroborate; 5: Polyoxyl 40 hydrogenated castor oil; 6: Dipropyl disulphide; 7: 2-Methyl-1-

pentanol; 8: Diethyl tolumide; 9: 1,4-Dibutoxy benzene; 10: 2,4,11,13-Tetraazatetradecane diimidamide, N,N''-bis(4chlorophenyl)-3,12-diimino-, di-D-gluconate; 11: Gamma-butyrolactone; 12: (Ethylenediamine-propyl)-trimethoxysilane; 13: Tetraethylene glycol diacrylate; 14: 1,2-Benzisothiazol-3(2H)-one; 15: 3,5-Dimethyl-2,5-hexanediol; 16: Disodium 2,2'-([1,1'-biphenyl]-4,4'diyldivinylene)bis-(benzenesulphonate); 17: Sodium oxalate; Cat1, serious damage; Cat2, mild irritant to irritant; NC, no damage.
